# Substance abuse in first-episode schizophrenic patients: a retrospective study

**DOI:** 10.1186/1745-0179-2-4

**Published:** 2006-03-23

**Authors:** MC Mauri, LS Volonteri, IF De Gaspari, A Colasanti, MA Brambilla, L Cerruti

**Affiliations:** 1 Clinical Psychiatry, Neuropsychopharmacology Unit, University of Milan, IRCCS Fondazione Ospedale Maggiore Policlinico, Via F.Sforza 35, 20122 Milano, Italy

## Abstract

Several studies suggest a high comorbidity of substance abuse and schizophrenia, associated with higher frequency of relapse, more positive symptoms and depression, cognitive impairment, poorer outcome and treatment response. A high incidence of substance abuse is also observed in first-episode patients. Among patients with substance abuse, the onset precedes the onset of psychosis of several years in most cases.

All the patients with a first episode of schizophrenia, at first admission to the Psychiatric Service of Diagnosis and Treatment of Ospedale Maggiore of Milan during the years 1990 to 2004, have been included in our study.

The clinical evaluation has been obtained considering the following items of Brief Psychiatric Rating Scale (BPRS): conceptual disorganization, depressed mood, hostility, hallucinations, unusual content of thought.

The results showed that 34.7% of first-episode schizophrenic patients had a lifetime history of substance abuse. The age of onset of schizophrenia is significantly lower for drug abusers than for patients without any type of abuse and for alcohol abusers (p < 0.005). In multi drug abusers, cannabis resulted the most frequently used (49%), followed by alcohol (13%), and cocaine (4%). Substance abusers have obtained a significant higher score in "thought disturbance" item (p < 0.005) and in "hostility" item (p < 0.005) compared to non substance abusers. Non drug abusers showed lower mean scores of "hostility" item compared to cocaine abusers and multi drug abusers (p < 0.005).

Our findings seem to indicate that substance abuse in the early course of illness determines an earlier onset of schizophrenia and increases severity of some psychotic symptoms like "hallucination" and "unusual content of thought". Therefore persons incurring a risk of schizophrenia may be warned of the possible relation between substances and psychosis and have to be counselled against the use of them.

## Introduction

A number of studies suggest a high level of comorbidity between substance abuse and schizophrenia [1-3], and approximately half of the patients suffering from schizophrenia have also been substance abusers at some point during their illness [4]. The comorbidity of schizophrenia and substance abuse is associated with more frequent relapses, more positive symptoms and depression, cognitive impairment, and a poorer outcome and treatment response [5-9].

Several hypotheses have been put forward to explain the relationship between schizophrenia and substance abuse [8,10-12]: it has been hypothesised that substance abuse may increase the risk of schizophrenia, at least in vulnerable individuals [13,14]; the self-medication hypothesis suggests that patients abuse drugs to alleviate the symptoms of psychosis or the debilitating side effects caused by antipsychotic medications, such as extrapyramidal symptoms [15]; and, finally, it could be a merely coincidental association of two psychiatric disorders that have similar age peaks in the distribution of onset and prevalence but without any causal interrelation.

A high incidence of substance abuse is also observed in first-episode patients [8], among whom the rates of substance abuse have been found to range from 20% to 30% [8,16]. In 2001 Sevy and coworkers found that the 23% of a sample of 118 patients with first-episode schizophrenia or schizoaffective disorder met the criteria for substance abuse or dependence disorder [5]. Among patients with substance abuse or dependence, the onset of substance abuse precedes the onset of psychosis by several years in most cases, and only in a minority begins at the time of a first episode or later [5,17]. Silver and Abboud found that 60% of first-admission schizophrenic patients had started substance abuse before their first hospitalisation [12].

In a large retrospective study [11], Allebeck observed that the onset of cannabis abuse preceded the onset of psychotic symptoms by at least one year in 69% of cases, and Rabinowitz also found that most of their patients had started substance abuse several years before the onset of psychosis [18].

Sevy and coworkers observed that alcohol and cannabis were the main substances for most first-episode patients [5]. Hambrecht and Hafner found that 13% of a sample of 232 first-episode schizophrenic patients had a history of cannabis abuse [2], and Linszen observed that 26% of a sample of recent-onset schizophrenic patients were cannabis abusers [17].

Various data indicate a relationship between cannabis use and schizophrenia, but its precise nature remains controversial [2,19]. The etiological hypothesis suggests that the use of cannabis may cause schizophrenia in vulnerable subjects (genetically or otherwise), makes its own contribution to the risk of becoming schizophrenic [2,19]. The vulnerability hypothesis is supported by the fact that substance abuse precedes the onset of psychosis in many patients, and that substance abusing patients are younger at the time of onset of schizophrenia. Hambrecht and Hafner found an earlier onset of psychosis among substance (mainly cannabis) abusers in one-third of their schizophrenic patients [8], and similar results have been found by Linszen [17], Buhler [20] and Maremanni [21].

The aim of this study was to identify the prevalence and pattern of substance abuse in first-episode schizophrenic patients, and the relationships between age of onset, demographic variables, pattern of symptomatology and other variables. Particular attention was given to cannabis use in relation to the onset of schizophrenia.

## Materials and methods

This study included all of the patients evaluated at the time of first admission to the Psychiatric Diagnosis and Treatment Service (SPDC) of Ospedale Maggiore, Milan, between the years 1990 and 2004 with a first episode of schizophrenia and comorbidity for substance abuse disorder or substance dependence following the DSM-IV-TR criteria. The patients were retrospectively diagnosed as being affected by schizophrenia on the basis of the DSM IV criteria during subsequent hospitalisations or outpatient visits.

A history of drug abuse was obtained from the information given by the patients and their relatives: the data concerned the type of abused substance, the age of onset of drug abuse, the pattern of drug abuse (before, during or after the onset of schizophrenia). Urine toxicology was performed if clinically required.

The subjects assessed as having a schizophrenia-like disorder and substance abuse upon admission were followed up during their hospitalisation after discontinuing substance use in order to confirm the diagnosis of schizophrenia. Subjects with a confirmed diagnosis of substance-induced psychosis were excluded from the study; the diagnosis was retrospectively formulated on the basis of subsequent observations during hospitalisations or outpatient visits according to the DSM IV criteria.

The study exclusion criteria were a previous diagnosis of schizophrenia, a previous diagnosis of substance-induced psychosis, a previous admission to any psychiatric hospital, steady treatment in any psychiatric service preceding admission to our SPDC, the taking of clozapine or any neuroleptic "depot" therapy, lifelong antipsychotic treatment for longer than 12 weeks, and the presence of an organic brain syndrome or severe mental retardation.

The clinical evaluation took into account the conceptual disorganisation, depressed mood, hostility, hallucinations and unusual thought content items of the Brief Psychiatric Rating Scale (BPRS) [22], and referred to the first admission to the ward; the clinical information was retrospectively obtained by consulting the medical records.

The data were statistically analysed by means of descriptive methods, analysis of variance (ANOVA), the chi-squared test, multifactor analysis of variance, Fisher's test (LSD), and regression analysis (simple regression), using Statgraphics plus 5 programs (2000 Manugistics, Inc. USA).

## Results

The results of this study indicate that 285 patients with schizophrenia underwent first admission to the SPDC of Milan's Ospedale Maggiore between 1990 and 2004: 180 men (62.2%) and 105 women (37.8%). Their mean age ± SD at the time of the onset of schizophrenic symptoms was 26.14 ± 6.58 years (range 15–43), and their mean age at first admission was 27.53 ± 6.44 years (range 15–44). The mean duration of hospitalisation was 17.4 ± 13.43 years (range 1–108).

**Figure 1:**
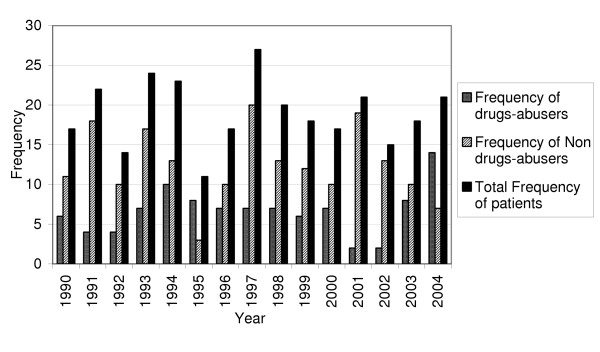
Frequency of hospitalized patients with the diagnosis of schizophreniform disease from 1990 to 2004.

Of these first-episode schizophrenic patients, 34.7% had a lifetime history of substance abuse: the distribution of the frequency of the schizophrenic patients on the basis of the presence/absence of substance abuse from 1990 to 2004 is shown in Figure 1. The substance abusers were significantly younger (24.99 ± 6.69 *vs *26.75 ± 6.45 years) (p < 0.05). There was a statistically significant difference in the gender distribution of substance abusers: 18.10% of the female schizophrenic patients were substance abusers, as against 44.44% of the male patients (p < 0.0001).

**Figure 2:**
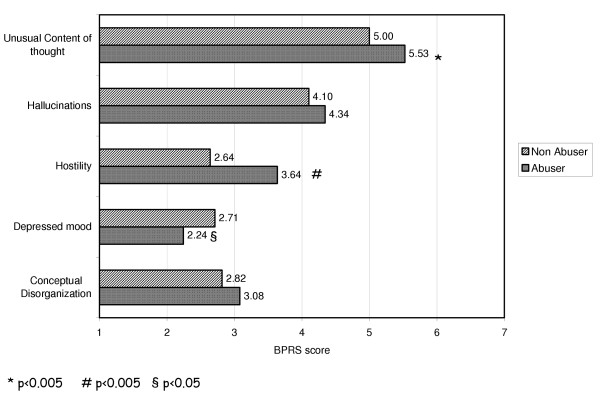
BPRS score in drug-abuser patients vs non drug-abuser.

From the clinical point of view, the substance abusers had significantly higher scores for the "unusual thought content" item (5.5 ± 1.53 *vs *5.0 ± 1.51; p < 0.005) and "hostility" item (3.63 ± 2.16 *vs *2.64 ± 1.84; p < 0.005), but significantly lower scores for the "depressed mood" item (2.24 ± 1.53 *vs *2.71 ± 1.67; p < 0.05) (Fig. 2).

**Figure 3:**
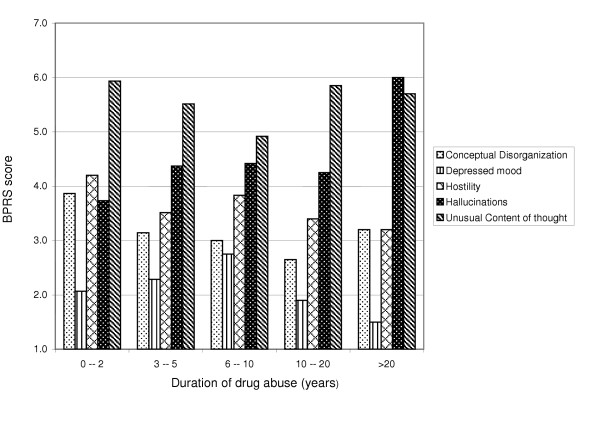
BPRS score related to the duration of drug abuse.

The people who had abused drugs for more than 20 years had significantly higher scores for the "hallucination" item than those who had abused drugs for a shorter time (Fig. 3).

**Figure 4:**
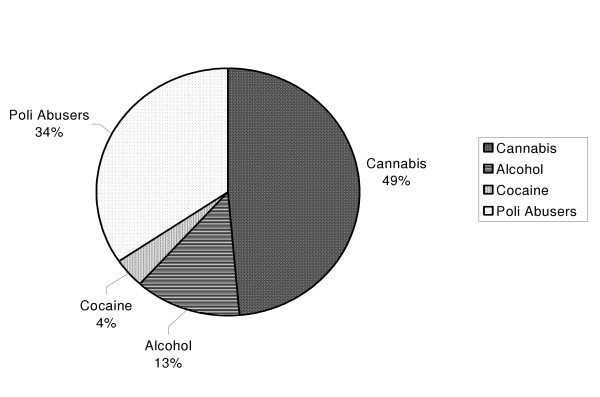
Percentage of drug used by the schizophreniform patients.

Among the abusers, 44% had taken only one substance throughout their life, whereas 56% were multi-drug abusers (the lifetime abuse of two or more substances, regardless of whether they were taken at the same time). The substances most frequently used by the single-substance abusers were cannabis (49%), followed by alcohol (13%) and cocaine (4%); among the multi-drug abusers, 38.24% used cannabis and cocaine, 35.29% cannabis and alcohol, 11.76% alcohol and hallucinogens, 5.88% cannabis and opioids, 5.88% alcohol and opioids, and 2.94% cannabis and amphetamines. The abuse of opioids and hallucinogens has therefore always been associated with another substance (cannabis or alcohol). Considering the drug abusers as a whole, cannabis was used by 80% of the patients, 81% of whom were men and 19% were women. The distribution of abused substances is shown in Figure 4.

The age of onset of schizophrenia was significantly lower among the cannabis abusers in comparison with both the cannabis non-abusers (23.97 ± 6.30 *vs *26.75 ± 6.45 years; p < 0.05) and the alcohol abusers (23.97 ± 6.30 *vs *29.84 ± 8.43 years; p < 0.05).

**Figure 5:**
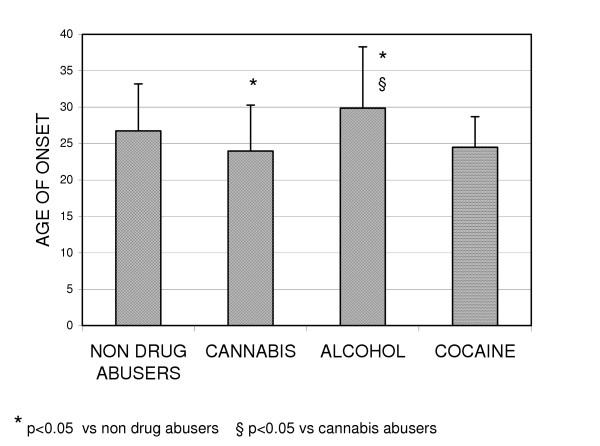
Age of onset of schizophrenia in the different diagnostic categories.

The mean age at first admission of cannabis abusers was significantly lower than that of the non-abusers (25.47 ± 6.10 *vs *27.98 ± 6.31 years; p < 0.005), whereas the mean age at first admission of the alcohol abusers was significantly higher than that of both the cannabis non-abusers (32.46 ± 8.05 *vs *27.98 ± 6.31 years; p < 0.005) and cannabis abusers (32.46 ± 8.05 *vs *25.47 ± 6.10 years; p < 0.005) (Fig. 5).

**Figure 6:**
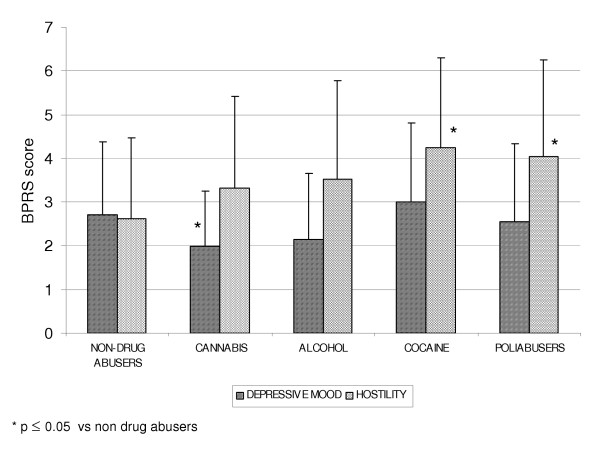
BPRS score : depressive mood and hostility in the different diagnostic categories.

The mean "hostility" item scores were significantly different between the non-drug abusers, the abusers of only one substance (cannabis, alcohol or cocaine), and the multi-drug abusers. In particular, the non-abusers had lower mean scores than the cocaine and multi- drug abusers (2.63 ± 1.84 *vs *4.25 ± 2.06 and 4.05 ± 2.21; p < 0.005) (Fig. 6). The mean "depressed mood" scores tended to be lower among cannabis abusers (1.97 ± 1.27) than among multi-drug abusers (2.55 ± 1.79), non-abusers (2.70 ± 1.67), alcohol abusers (2.15 ± 1.51) and cocaine abusers (3.0 ± 1.82) (p ≤ 0.05) (Fig. 6).

In relation to the temporal relationship between substance abuse and the first schizophrenic symptoms, the former preceded the onset of schizophrenia in 98.97% of the cases, and the mean duration of substance abuse before the first symptoms was 7.07 ± 5.73 years (range 1–29). In the remaining 2.03%, substance abuse had begun at the same time as the appearance of the first clinical symptoms (± 6 months); all of these cases concerned cannabis use.

There was no statistical difference between the patients with and without a history of substance abuse in terms of a family history of psychiatric disorders.

The mean duration of hospitalisation was not statistically different between the substance abusers and non-abusers, but was significantly longer among the multi-drug abusers than the single drug abusers (16.03 ± 11.65 *vs *23.05 ± 12.34 days; p = 0.006).

## Discussion

Our results suggest a high prevalence of substance abuse (37.4%) in first-episode schizophrenia in a sample of patients undergoing first admission. This is in line with published data indicating a 35% lifetime prevalence of drug abuse among first-episode cases of schizophrenia [5,20,23,24], and our finding of a higher proportion of male patients among the substance abusers is also in line with those of previous studies [5,8,18,24].

As reported in most previous studies [5,8,18,25], the most frequently abused substance in our first-onset schizophrenic patients was cannabis, followed by alcohol. The frequency of substance (particularly cannabis) abuse is in line with recent general population data. The use of cannabis seems to be more frequent among young people [26] and its cannabinoid content has increased enormously over the last 20 years [27]. In a cohort study of 2032 students, Patton found that 66% of the males and 52% of the female participants reported having used cannabis at some point, and 75% had started to use cannabis when they were teeenagers [28]. Similar findings have been reported by other authors in different countries [29,30].

Our findings seem to indicate that substance abuse leads to an earlier onset of schizophrenia as the age at onset of schizophrenia was significantly lower among the drug abusers than among the patients without any type of abuse or alcohol abusers. This is in line with the findings of several previous studies [11,20,31,32].

The start of substance abuse preceded the onset of schizophrenia by several years in most of our patients, which seems to contradict the self-medication hypothesis. However, as pointed out by other authors, it is possible that substance use improves some latent symptoms experienced by the patients before the onset of schizophrenia [5,11,12].

In line with the observations of Sevy [5] and Andreasson [32], we did not find any difference in the family histories of schizophrenic substance abusers or non- abusers. These purely anamnestic data seem to support an association between substance abuse and schizophrenia due to genetic vulnerability, and do not seem to confirm the observations of McGuire and coworkers who found a particularly high risk of schizophrenia among the relatives of probands who developed, or relapsed into, psychosis in the context of cannabis use [33].

However, the temporal sequence and younger age of onset in the group of abusers suggest that schizophrenia onsets may be precipitated by the start of drug abuse, particularly the abuse of cannabis. One biological explanation of the relationship between cannabis abuse and the onset of schizophrenia may be found in recent pharmacological and genetic studies [34-36]. Molecular studies suggest that cannabis can increase mesolimbic dopaminergic transmission and inhibit glutamatergic release, in line with current biological theories concerning schizophrenia [37]. The recently suggested role of the endogenous cannabinoid system in schizophrenia unrelated to previous cannabinoid consumption introduces an additional perspective concerning the mechanism underlying cannabis-associated schizophrenia [38].

Studies of dual-diagnosis patients also suggest that substance abuse has an effect on symptom presentation. In comparison with non-abusing schizophrenic patients, dual-diagnosis patients have been found to be more depressed, more thought-disordered, and to have more positive and negative symptoms [39,40].

Our results seem to confirm that substance abuse during the early course of illness increases the severity of some psychotic symptoms such as "hallucinations" and "unusual thought content". We found that first-episode patients with a history of substance abuse have more thought disturbances, hallucinations and hostility than those with no such history. Hostility is particularly increased in patients who abuse cocaine or more than one substance, possibly as an action of the substances themselves favouring or worsening the appearance of psychotic disorders. There have been suggestions that depressed individuals are more likely to use cannabis, and that the consumption of cannabis is associated with an increase in anxiety, depression and suicide attempts [41]; however, we found that our cannabis abusing patients tended to be less depressed, although this may be due to various difficult-to-investigate variables, such as the duration of abuse, the time since the substance was last taken, and the amount of cannabis used.

In conclusion, our study indicates that drug (particularly cannabis) abuse is frequent among patients with first-episode schizophrenia, and has a considerable effect on symptom presentation; it also seems to precipitate the onset of schizophrenia at an earlier age. These findings, which are in line with other recently published data, underline the need to act early on cannabis abuse. People at risk of schizophrenia should be warned of the possible relationship between cannabis and psychosis, and counselled against the use of cannabis.
